# Supplementary material for: Mitochondria dysfunction in Charcot Marie Tooth 2B Peripheral Sensory Neuropathy
Source: Commun Biol. 2022 Jul 18;5:717. doi: 10.1038/s42003-022-03632-1 (PMC9293960; doi:10.1038/s42003-022-03632-1)
Supplement: Supplementary file 1 — Supplementary Information [file 42003_2022_3632_MOESM1_ESM.pdf]

Supplemental Fig 1. A CMT2B Rab7^{V162M} knockin mouse model (C57BL6)

a: a schematic shows the knockin strategy. b: PCR primers and tail DNA genotyping results of wt (+/+), heterozygote (fl/+) and homozygote (fl/fl). c: both fl/+, fl/fl mice survived to adulthood.

Supplemental Fig 2. Mitochondrial morphology in soma of DRG sensory neurons

a: Representative images of Mito Tracker in E18 DRG sensory neurons from +/+, fl/+, fl/fl embryos. b: The phase contrast images corresponding to a.

Supplemental Fig 3. Mitochondrial morphology in soma of hippocampal neurons

a: Representative images of Mito Tracker in E18 hippocampal neurons from +/+, fl/+, fl/fl embryos. b: The phase contrast images corresponding to a.

Supplemental Fig 4. Mitochondrial morphology in soma of cortical neurons

a: Representative images of Mito Tracker in E18 cortical neurons from +/+, fl/+, fl/fl embryos. b: The phase contrast images corresponding to a.

Supplemental Fig 5. Raw images for Western blots in Fig 1, Fig 7

a: Raw images of blotting pDrp1-S616 and total Drp1in human fibroblasts. b: Raw images of blotting Drp1, TOM20 and beta-Actin in MEFs.

Supplemental Movie s1. Axonal trafficking of mitochondria in DRG sensory neurons from +/+ E18 embryos

Supplemental Movie s2. Axonal trafficking of mitochondria in DRG sensory neurons from fl/+ E18 embryos

Supplemental Movie s3. Axonal trafficking of mitochondria in DRG sensory neurons from fl/fl E18 embryos

a: Knockin Strategy

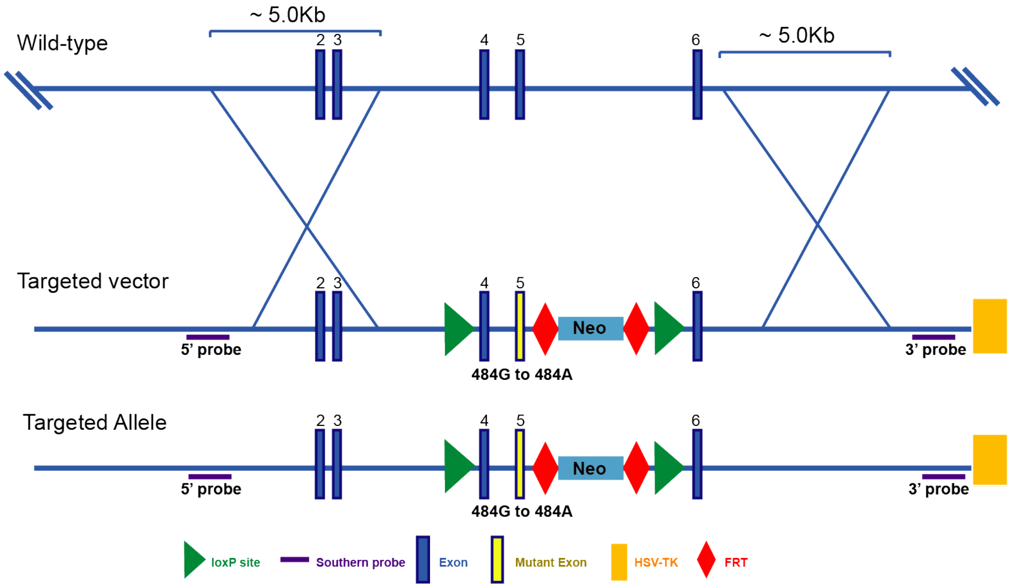

b: Genotyping

Primer 1 (Rab7-loxPtF2)
GAGCTCCTGGAATTTGAGATTATG
Primer 2 (Rab7-loxPtR2)
TAACAGGGCACTCCCCAATACAGA

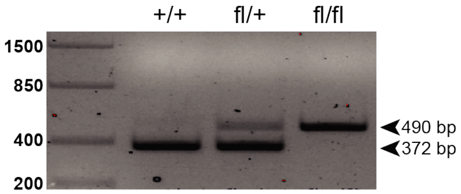

c: Survival

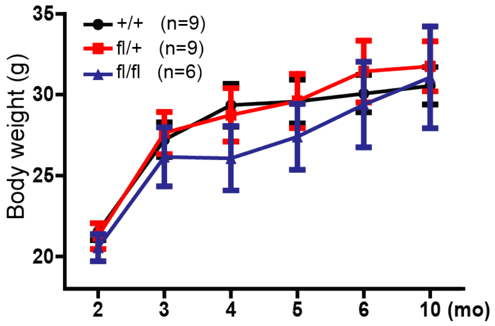

Supplemental Fig 1. A CMT2B Rab7^{V162M} knockin mouse model (C57BL6)

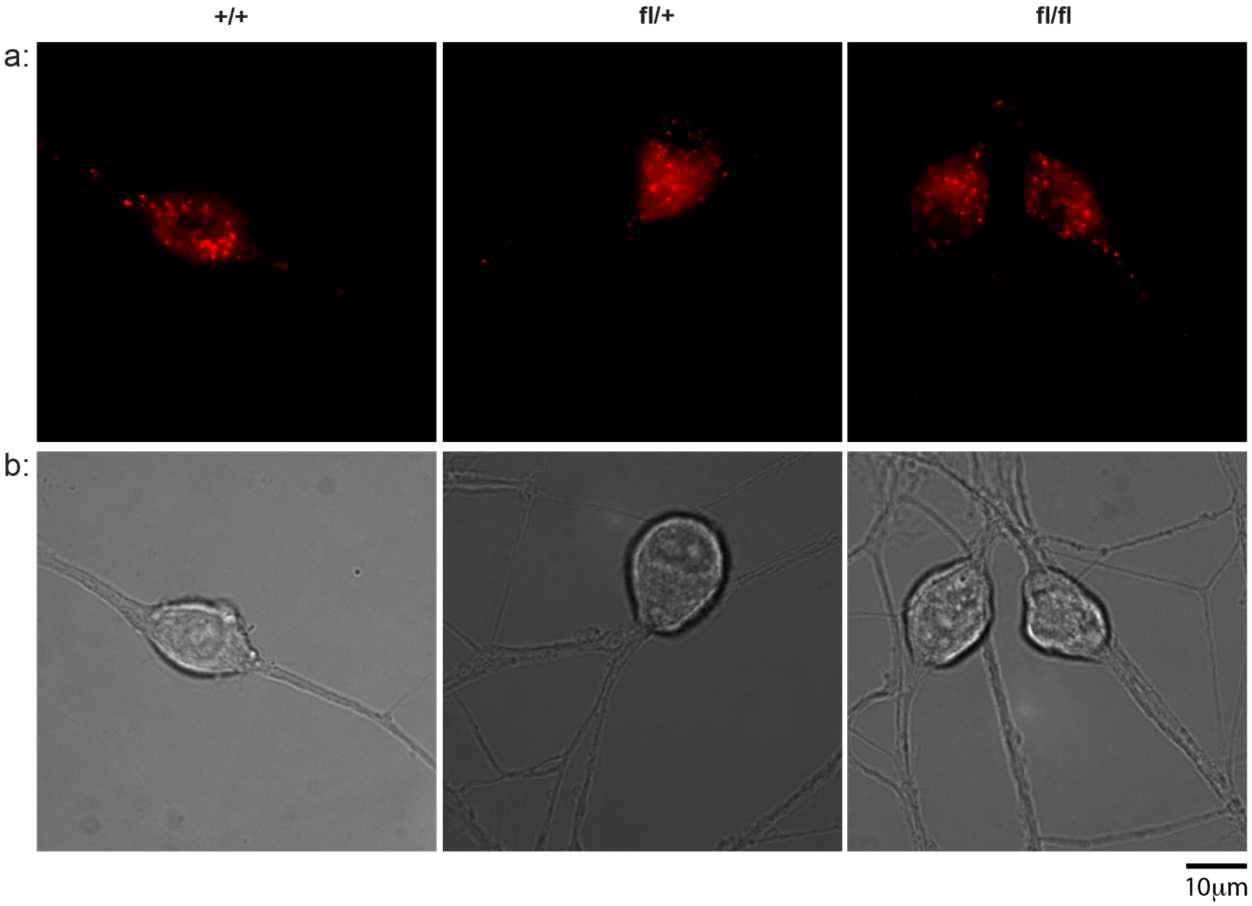

Supplemental Fig 2. Mitochondrial morphology in soma of DRG sensory neurons

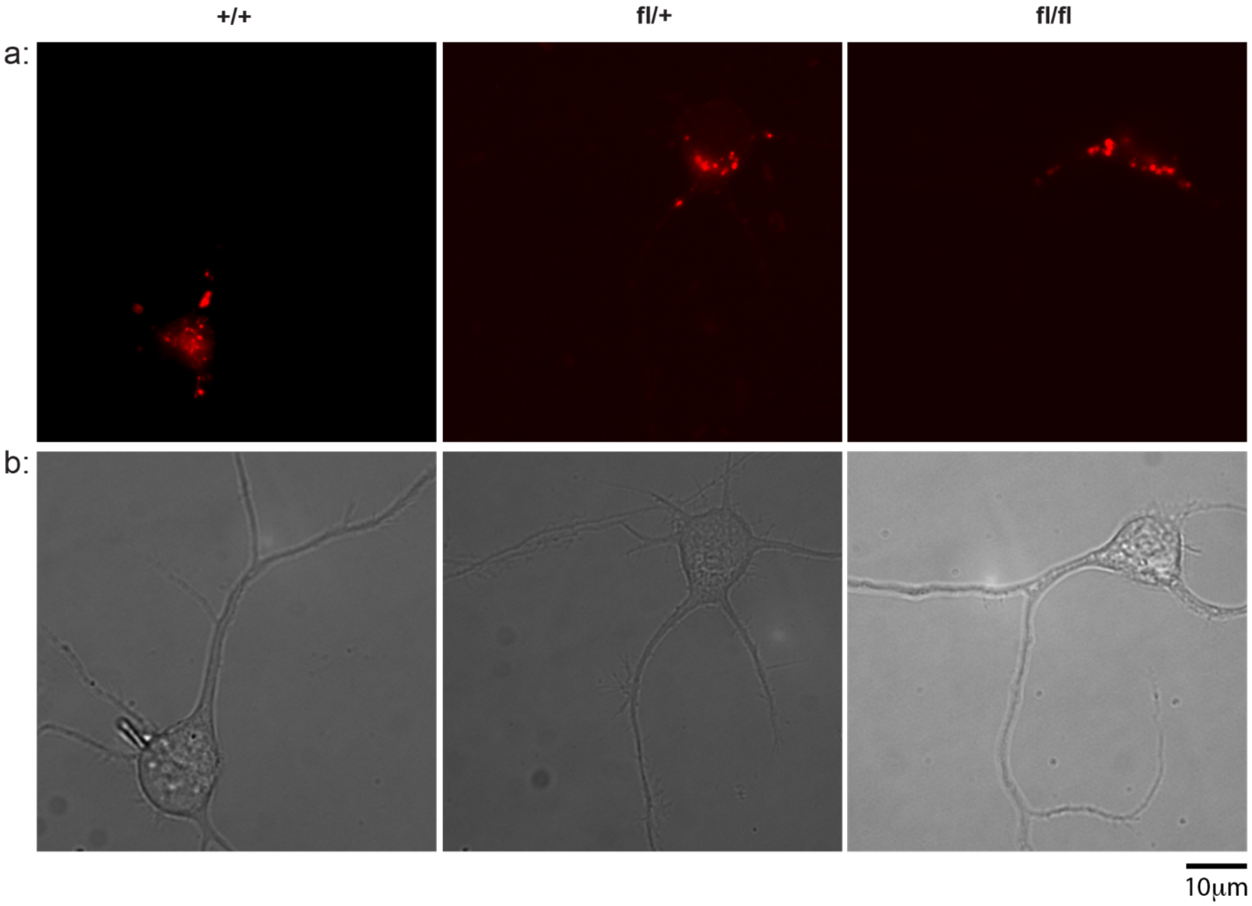

Supplemental Fig 3. Mitochondrial morphology in soma of hippocampal neurons

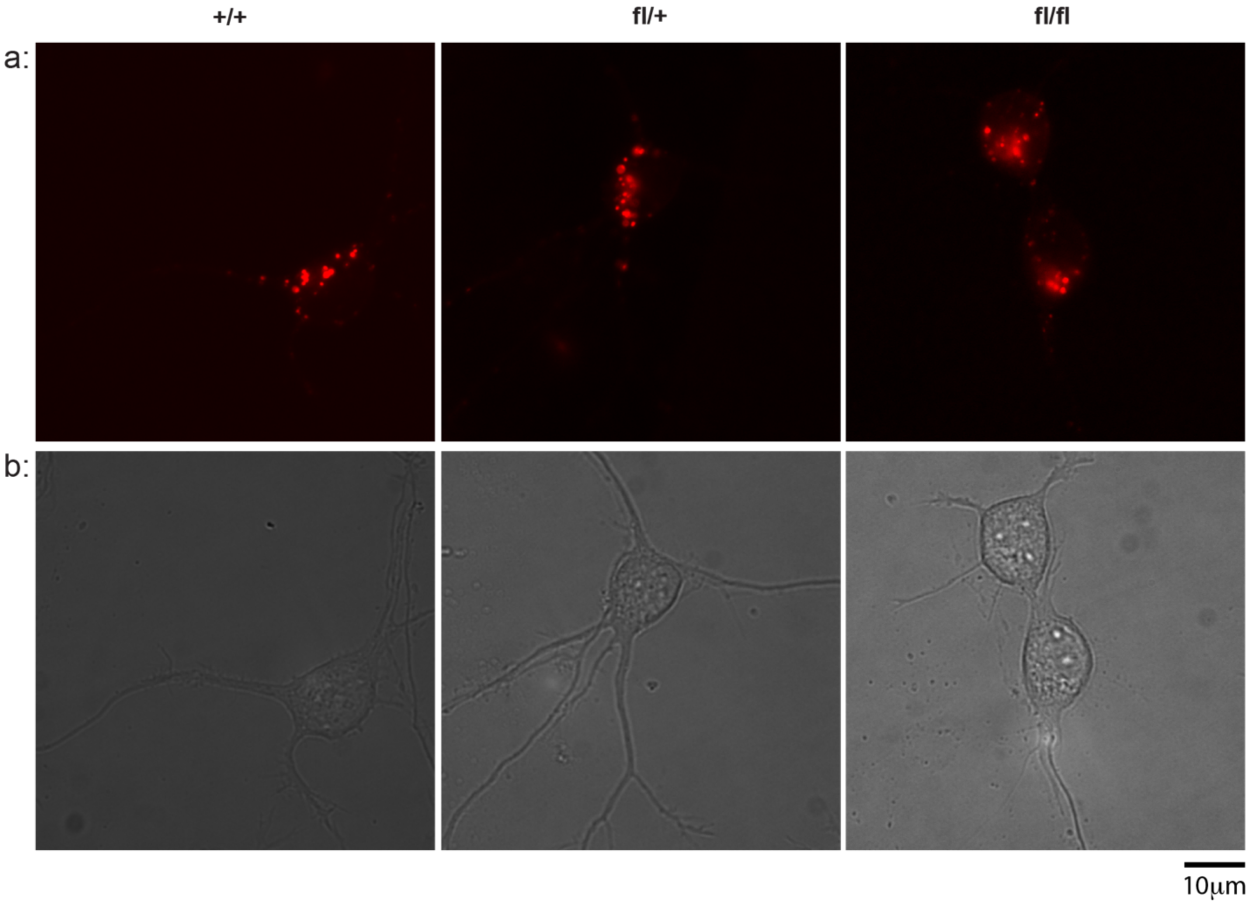

Supplemental Fig 4. Mitochondrial morphology in soma of cortical neurons

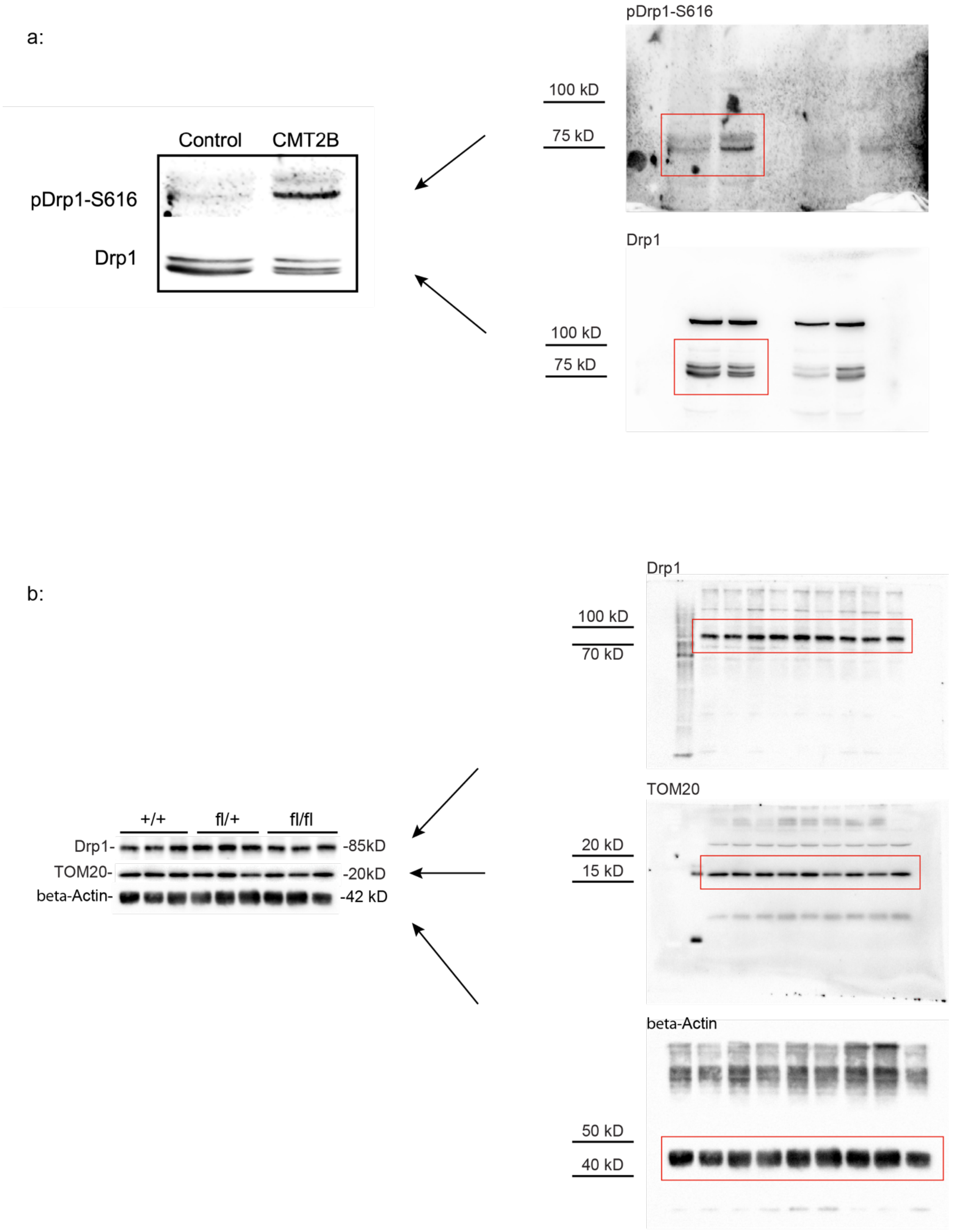

Supplemental Fig 5. Raw images for Western blot
