# Supplementary material for: Mitochondria dysfunction in Charcot Marie Tooth 2B Peripheral Sensory Neuropathy
Source: Commun Biol. 2022 Jul 18;5:717. doi: 10.1038/s42003-022-03632-1 (PMC9293960; doi:10.1038/s42003-022-03632-1)
Supplement: Supplementary file 2 — Description of Additional Supplementary Files [file 42003_2022_3632_MOESM2_ESM.pdf]

Description of Additional Supplementary Files

File name: Movie S1
Description: Axonal trafficking of mitochondria in DRG sensory neurons from +/+ E18 embryos.

File name: Movie S2
Description: Axonal trafficking of mitochondria in DRG sensory neurons from fl/+ E18 embryos.

File name: Movie S3
Description: Axonal trafficking of mitochondria in DRG sensory neurons from fl/fl E18 embryos.
