# Supplementary material for: Mitochondria dysfunction in Charcot Marie Tooth 2B Peripheral Sensory Neuropathy
Source: Commun Biol. 2022 Jul 18;5:717. doi: 10.1038/s42003-022-03632-1 (PMC9293960; doi:10.1038/s42003-022-03632-1)
Supplement: Supplementary file 6 — Reporting Summary [file 42003_2022_3632_MOESM6_ESM.pdf]

nature portfolio

Corresponding author(s): Chengbiao Wu; Cecilia Bucci

Last updated by author(s): May 8, 2022

Reporting Summary

Nature Portfolio wishes to improve the reproducibility of the work that we publish. This form provides structure for consistency and transparency in reporting. For further information on Nature Portfolio policies, see our Editorial Policies and the Editorial Policy Checklist.

Statistics

For all statistical analyses, confirm that the following items are present in the figure legend, table legend, main text, or Methods section.

n/a	Confirmed	
[ ]	[x]	The exact sample size (*n*) for each experimental group/condition, given as a discrete number and unit of measurement
[ ]	[x]	A statement on whether measurements were taken from distinct samples or whether the same sample was measured repeatedly
[ ]	[x]	The statistical test(s) used AND whether they are one- or two-sided<br>*Only common tests should be described solely by name; describe more complex techniques in the Methods section.*
[x]	[ ]	A description of all covariates tested
[ ]	[x]	A description of any assumptions or corrections, such as tests of normality and adjustment for multiple comparisons
[ ]	[x]	A full description of the statistical parameters including central tendency (e.g. means) or other basic estimates (e.g. regression coefficient) AND variation (e.g. standard deviation) or associated estimates of uncertainty (e.g. confidence intervals)
[ ]	[x]	For null hypothesis testing, the test statistic (e.g. *F*, *t*, *r*) with confidence intervals, effect sizes, degrees of freedom and *P* value noted<br>*Give P values as exact values whenever suitable.*
[x]	[ ]	For Bayesian analysis, information on the choice of priors and Markov chain Monte Carlo settings
[ ]	[x]	For hierarchical and complex designs, identification of the appropriate level for tests and full reporting of outcomes
[x]	[ ]	Estimates of effect sizes (e.g. Cohen's *d*, Pearson's *r*), indicating how they were calculated

Our web collection on statistics for biologists contains articles on many of the points above.

Software and code

Policy information about availability of computer code

Data collection	Leica DMi8 Live Imaging Microscope; Confocal laser scanning microscope (CLSM) (Zeiss, LSM 700, Germany) equipped with a laser diode emitting at 405nm, an argon-ion laser for excitation at 488nm, and a helium-neon laser for excitation at 555nm. Plan-Apochromat 63.0 × 1.40 oil-immersion objective DIC M27; ZEN Black Edition 2011 software; ECL-Clarity (BioRad); ChemiDoc XRS+ (BioRad); ImageLab 6.0.1 software (BioRad)
Data analysis	NIH ImageJ (Fiji) software; Mitochondria Analyzer plugin was downloaded from http://sites.imagej.net/ACMito/

For manuscripts utilizing custom algorithms or software that are central to the research but not yet described in published literature, software must be made available to editors and reviewers. We strongly encourage code deposition in a community repository (e.g. GitHub). See the Nature Portfolio guidelines for submitting code & software for further information.

Data

Policy information about availability of data

All manuscripts must include a data availability statement. This statement should provide the following information, where applicable:

- Accession codes, unique identifiers, or web links for publicly available datasets
- A description of any restrictions on data availability
- For clinical datasets or third party data, please ensure that the statement adheres to our policy

The datasets generated during and/or analysed during the current study are available from the corresponding author on reasonable request.

Field-specific reporting

Please select the one below that is the best fit for your research. If you are not sure, read the appropriate sections before making your selection.

☒ Life sciences ☐ Behavioural & social sciences ☐ Ecological, evolutionary & environmental sciences

For a reference copy of the document with all sections, see nature.com/documents/nr-reporting-summary-flat.pdf

Life sciences study design

All studies must disclose on these points even when the disclosure is negative.

Sample size	no sample size calculation was performed since our sample size is large enough to meet the statistical anslysis.
Data exclusions	no data was excluded from the analysis.
Replication	human and animal experiments were carried out respectively and independently by Universita del Salento and UCSD, and we obtained the consistent results. Additionally, each part of animal experiment was conducted at least three times.
Randomization	in investigating the effect of Mdivi-1 and/ or CID treatment, the cells were allocated into control and series of treatment groups according to the numbers sequence on the culture plates.
Blinding	both the analyzers and data collectors were blinded to group allocation.

Reporting for specific materials, systems and methods

We require information from authors about some types of materials, experimental systems and methods used in many studies. Here, indicate whether each material, system or method listed is relevant to your study. If you are not sure if a list item applies to your research, read the appropriate section before selecting a response.

Materials & experimental systems

n/a	Involved in the study
☐	☒ Antibodies
☒	☐ Eukaryotic cell lines
☒	☐ Palaeontology and archaeology
☐	☒ Animals and other organisms
☐	☒ Human research participants
☒	☐ Clinical data
☒	☐ Dual use research of concern

Methods

n/a	Involved in the study
☒	☐ ChIP-seq
☒	☐ Flow cytometry
☒	☐ MRI-based neuroimaging

Antibodies

Antibodies used	Rabbit monoclonal Ab against Drp1 (D6C7) was from Cell Signaling Technology (CST#8570S), rabbit anti-TOM20 (sc-11415) and mouse anti-β-Actin were from Santa Cruz Biotechnology. Primary antibodies against human phospho(p)-Drp1 Ser616 (cat#3455) were from Cell Signaling (Danvers, MA, USA) and against total Drp1 (sc.271583) from Santa Cruz Biotechnologies (Dallas, TX, USA). Goat anti-rabbit and anti-mouse IgG-HRP conjugates were purchased from Jackson ImmuoResearch Laboratories.
Validation	DRP1 (D6C7) Rabbit mAb recognizes endogenous levels of total DRP1 protein. Species Reactivity: Human, Mouse, Rat, Monkey.<br>Anti-Tom20 Antibody is a mouse monoclonal IgG2a λ Tom20 antibody, cited in 473 publications, raised against amino acids 1-145 of Tom20 human origin. Anti-Tom20 Antibody is recommended for detection of Tom20 of mouse, rat and human origin by WB, IP, IF, IHC(P) and ELISA.<br>Phospho-DRP1 (Ser616) Rabbit Antibody detects endogenous levels of DRP1 only when phosphorylated at Ser616. Species Reactivity: Human.<br>Anti-DRP1 Antibody (sc-271583) is a mouse monoclonal IgG1 κ DRP1 antibody, cited in 94 publications, raised against amino acids 560-736 mapping at the C-terminus of DRP1 of human origin. Anti-DRP1 Antibody is recommended for detection of all DRP1 isoforms of mouse, rat and human origin by WB, IP, IF, IHC(P) and ELISA.

Animals and other organisms

Policy information about studies involving animals; ARRIVE guidelines recommended for reporting animal research

Laboratory animals	mouse, C67BL6, female and male, embryos
Wild animals	no relevant

Field-collected samples	no relevant
Ethics oversight	All experiments involving the use of laboratory animals have been approved by the Institutional Animal Care and Use Committee of University of California San Diego. Surgical and animal procedures were carried out strictly following the NIH Guide for the Care and Use of Laboratory Animals.

Note that full information on the approval of the study protocol must also be provided in the manuscript.

Human research participants

Policy information about studies involving human research participants

Population characteristics	All experiments involving the use of laboratory animals have been approved by the Institutional Animal Care and Use Committee of University of California San Diego. Surgical and animal procedures were carried out strictly following the NIH Guide for the Care and Use of Laboratory Animals.
Recruitment	The patients who meet the diagnostic criteria of CMT2B were recruited. In order to avoid any potential self-selection bias, the patients with possible mitochondria involved diseases were excluded. Healthy individuals were age-matched.
Ethics oversight	Informed consent was obtained in compliance of the Helsinki Declaration. The Study was approved by the local Ethics Committee (Ethical Committee Approval Protocol # 107/05). All samples were anonymously encoded to protect patient confidentiality.

Note that full information on the approval of the study protocol must also be provided in the manuscript.
